# Diversity, Loss, and Gain of Malaria Parasites in a Globally Invasive Bird

**DOI:** 10.1371/journal.pone.0021905

**Published:** 2011-07-11

**Authors:** Alfonso Marzal, Robert E. Ricklefs, Gediminas Valkiūnas, Tamer Albayrak, Elena Arriero, Camille Bonneaud, Gábor A. Czirják, John Ewen, Olof Hellgren, Dita Hořáková, Tatjana A. Iezhova, Henrik Jensen, Asta Križanauskienė, Marcos R. Lima, Florentino de Lope, Eyðfinn Magnussen, Lynn B. Martin, Anders P. Møller, Vaidas Palinauskas, Péter L. Pap, Javier Pérez-Tris, Ravinder N. M. Sehgal, Manuel Soler, Eszter Szöllősi, Helena Westerdahl, Pavel Zetindjiev, Staffan Bensch

**Affiliations:** 1 Department of Biology, Lund University, Lund, Sweden; 2 Department of Anatomy, Cellular Biology and Zoology, University of Extremadura, Badajoz, Spain; 3 Department of Biology, University of Missouri, St. Louis, Missouri, United States of America; 4 Nature Research Centre, Vilnius, Lithuania; 5 Mehmet Akif Ersoy Üniversitesi, Fen Edebiyat Fakültesi, Biyoloji Bölümü, Burdur, Turkey; 6 Laboratoire de Parasitologie Evolutive, CNRS UMR7103, UPMC Univ Paris 06, Paris, France; 7 Station d'Ecologie Expérimentale du CNRS USR 2973, Moulis, France; 8 Department of Infectious Diseases, University of Agricultural Sciences and Veterinary Medicine, Cluj-Napoca, Romania; 9 Institute of Zoology, Zoological Society of London, London, United Kingdom; 10 Department of Zoology, Faculty of Science, Charles University in Prague, Prague, Czech Republic; 11 Department of Biology, Centre for Conservation Biology, Norwegian University of Science and Technology, Trondheim, Norway; 12 Departamento de Ecologia-IB, Pós-Graduação em Ecologia, Universidade de Brasília, Brasília, Brazil; 13 Faculty of Science and Technology, University of the Faroes, Tórshavn, Faroe Islands; 14 Department of Integrative Biology, University of South Florida, Tampa, Florida, United States of America; 15 Laboratoire d'Ecologie, Systématique et Evolution, CNRS UMR 8079, Université Paris-Sud, Bâtiment 362, Orsay, France; 16 Department of Taxonomy and Ecology, Babes-Bolyai University, Cluj-Napoca, Romania; 17 Department of Zoology and Physical Anthropology, Complutense University, Madrid, Spain; 18 Department of Biology, San Francisco State University, San Francisco, California, United States of America; 19 Department of Animal Biology, University of Granada, Granada, Spain; 20 Department of Systematic Zoology and Ecology, Eötvös Loránd University, Budapest, Hungary; 21 Central Laboratory of General Ecology, Bulgarian Academy of Sciences, Sofia, Bulgaria; 22 Centre of Ecology and Conservation, School of Biosciences, University of Exeter, Cornwall Campus, Cornwall, United Kingdom; 23 Center for Advanced Study, Oslo, Norway; Smithsonian Institution National Zoological Park, United States of America

## Abstract

Invasive species can displace natives, and thus identifying the traits that make aliens successful is crucial for predicting and preventing biodiversity loss. Pathogens may play an important role in the invasive process, facilitating colonization of their hosts in new continents and islands. According to the Novel Weapon Hypothesis, colonizers may out-compete local native species by bringing with them novel pathogens to which native species are not adapted. In contrast, the Enemy Release Hypothesis suggests that **flourishing** colonizers are successful because they have left their pathogens behind. To assess the role of avian malaria and related haemosporidian parasites in the global spread of a common invasive bird, we examined the prevalence and genetic diversity of haemosporidian parasites (order Haemosporida, genera *Plasmodium* and *Haemoproteus*) infecting house sparrows (*Passer domesticus*). We sampled house sparrows (N = 1820) from 58 locations on 6 continents. All the samples were tested using PCR-based methods; blood films from the PCR-positive birds were examined microscopically to identify parasite species. The results show that haemosporidian parasites in the house sparrows' native range are replaced by species from local host-generalist parasite fauna in the alien environments of North and South America. Furthermore, sparrows in colonized regions displayed a lower diversity and prevalence of parasite infections. Because the house sparrow lost its native parasites when colonizing the American continents, the release from these natural enemies may have facilitated its invasion in the last two centuries. Our findings therefore reject the Novel Weapon Hypothesis and are concordant with the Enemy Release Hypothesis.

## Introduction

Invasive species can have substantial impacts on ecological systems [1], [2], gene pools [3], and the disease environments for livestock, crops, and humans [4]. Still, intentional and unintentional introductions of non-indigenous species continue. Hence, understanding the causes of invasion success can help us to predict effects of species introductions and design interventions.

Range shifts of host or parasite species may result in species interactions not previously experienced in the wild [5]. Consequently, introduced species might encounter novel parasites whose virulence will partly determine whether they become invasive. We expect parasites with broader native host ranges to emerge in novel hosts more readily, but empirical data on this point are scarce because natural host ranges are unknown for most parasite species. Two main hypotheses have been developed to explain the success of invaders in their new environment. The *Novel Weapon Hypothesis* (NWH) proposes that introduced species possess allelopathic chemicals or pathogenic parasites against which native species have not evolved defenses [6], [7]. In contrast, the *Enemy Release Hypothesis* (ERH) suggests that non-native species become successfully established because they are freed from their co-evolved pathogens, parasites and predators [8]–[11].

More than 200 avian haemosporidian species have been described based on morphology among 4,000 investigated bird species [12]. The most commonly found blood parasites of birds belong to the genera *Plasmodium* and *Haemoproteus*. These parasites are transmitted exclusively by blood-sucking dipteran insects (Diptera). Mosquitoes (Culicidae) are vectors of *Plasmodium* species, while biting midges (Ceratopogonidae) and hippoboscid flies (Hippoboscidae) are the vectors of avian haemoproteids (Haemoproteidae) [12]. Vector abundance and/or diversity usually decrease towards higher latitudes [13], which could determine the presence or absence of appropriate vector-parasite-host interactions [14] and, consequently, colonization success. Surveys of the haemosporidian parasites of birds provide unprecedented knowledge of the host and geographic distributions of hundreds of parasite species or evolutionary lineages [15], thus presenting unique opportunities to investigate the origin of novel pathogens in invasive species. The facts that 1) birds are inter- and transcontinental migrants, 2) their haemosporidian parasites have worldwide distribution and 3) they often are host-generalists make this host-parasite model particularly attractive in addressing questions concerning transmission, specificity and speciation of parasites.

The house sparrow, *Passer domesticus* (Passeriformes: Passeridae), is native to the Mediterranean region and other areas with warm summer climates in Europe and Central Asia [16] from where it expanded its range into Northern Europe during the Bronze Age [17]. Over the last two centuries and with human assistance, the house sparrow spread to all continents (except Antarctica) and to many oceanic islands [18]. The avian malaria parasite *Plasmodium relictum*, common in European house sparrows [19], has been recorded in other avian species in all zoogeographical regions [12]. This morphospecies includes several mitochondrial cytochrome *b* (cyt *b*) gene lineages [20], one being the lineage GRW4 that has contributed to the decline and extinction of several endemic bird species following its introduction to Hawaii [21]. In Europe, Africa and Asia, several *P. relictum* lineages infect a wide range of passerine birds [15], [22]. The *P. relictum* lineage SGS1 has been reported to be prevalent among house sparrows in France [19] and an experimental infection study demonstrated that it is of low virulence in European house sparrows, but is highly virulent in several other passeriform host species [23]. Although of low virulence, a recent study showed that the lineage SGS1 exerts diversifying selection on the local composition of Major Histocompatibility Complex (MHC) alleles across populations of house sparrows [24].

Because of the current almost global distribution of *Plasmodium relictum*, it is possible that some of its lineages were spread along with the house sparrow [25] and have facilitated the colonization of house sparrows by reducing populations of susceptible native competitors. Alternatively, some native haemosporidian parasites might be more virulent to house sparrows than *P. relictum.* For instance, *Haemoproteus* parasites have been shown to reduce survival of house sparrows in several studies in Europe [26], [27]. If such harmful parasites were left behind when house sparrows colonized the new continents, this might have contributed to the rapid spread and increase in the new range. Few studies have previously tested the role of haemosporidian parasites in invasive birds, generally giving inconclusive results. For example, the prevalence of haemosporidian parasites did not differ significantly between Indian native and mainland introduced populations of common myna *Acridotheres tristis* in Africa and Australia [28], whereas the parasite prevalence of house sparrows in their native range was higher when compared to those introduced in Central Brazil [29].

**Figure 1:**
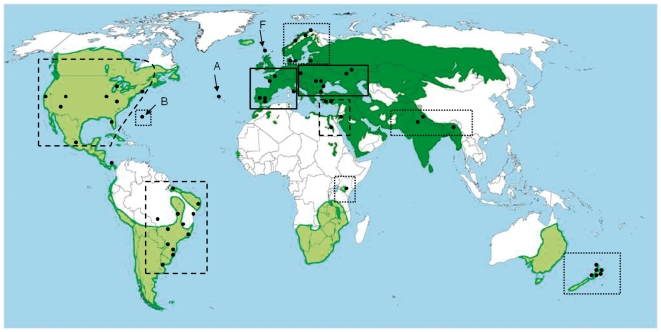
Geographical distribution and sampling sites of house sparrows. Dark green area shows natural range and light green introduced range. Sampling sites (black dots) and *Plasmodium* spp. lineage diversity within regions are illustrated by dotted lines (2–3 malaria parasite lineages), broken lines (4 malaria parasite lineages) and solid lines (8–9 malaria parasite lineages). The three sampled islands, Bermuda (B), Azores (A) and Faroe Islands (F), are indicated by arrows. The map is adapted from *Glutz von Blotzheim et al. (1997) and Anderson (2006)*

Here, we tested the potential contributions of the *Novel Weapon Hypothesis* (NWH) and the *Enemy Release Hypothesis* (ERH) to the successful invasion of house sparrows to new continents. We used blood samples of house sparrows from 58 localities on six continents (Fig. 1, Table S1) to characterize the identity and prevalence of haemosporidian parasites of the genera *Plasmodium* and *Haemoproteus*. Specifically we asked (a) whether this invasive species carried its parasites to new areas, acquired new local parasites, or both; and (b) whether the prevalences of infections differed between native and introduced areas and between old and newly-acquired parasites. If house sparrows retained their native parasite fauna in newly colonized regions, we could reject the ERH; if they replaced their native parasites with local parasites of the newly colonized continents, then we could reject the NWH, at least with respect to haemosporidian parasites. We found that house sparrow in newly colonized areas did not retain their native parasites, supporting the hypothesis that the release from these natural enemies facilitated its spread in the last two centuries.

## Results and Discussion

Of the 1820 house sparrows screened, 576 (31.6%) were infected by *Plasmodium* spp. and 87 (4.8%) by *Haemoproteus* spp. parasites. Only 56 (3.1%) house sparrows were found to harbour mixed infections. Although previous studies have found that mixed infections of blood parasites are common in wild birds [12], the low number of these infections might indicate that they decrease survival [30]. However, this finding should be interpreted with care because general PCR based methods tend to underestimate the frequency of mixed infections [31]. Sequencing the PCR positive samples revealed parasites of 30 different cyt *b* lineages, most of which have not been formally named and described based on morphological characters. Even though phylogenetic species limits have not been well defined in avian malaria species, cyt *b* lineages seem to represent reproductively isolated entities [32].

**Figure 2:**
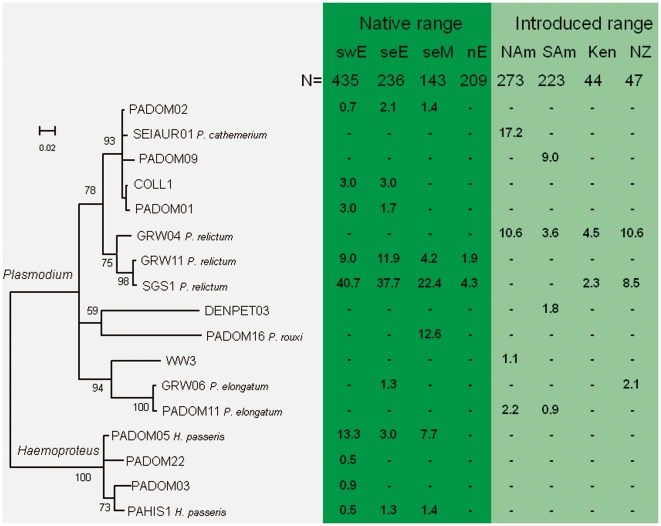
Phylogenetic relationships of *Plasmodium* and *Haemoproteus* cytochrome *b* lineages recorded in houses sparrows and their prevalence (%) in eight geographic regions. Regions with more than 40 sampled sparrows were included: South West Europe (swE), South East Europe (seE), South East Mediterranean (seM), North Europe (nE), North America (NAm), South America (SAm), Kenya (Ken) and New Zealand (NZ). Lineages recorded in single individuals were excluded (7 in Europe, 3 in N America, 2 in S America and 1 in India). The phylogeny was constructed from 479 bp cytochrome *b* sequences using Bayesian Inference and rooted with three lineages of *Leucocytozoon* (see Methods). Numbers at the nodes indicate the Bayesian posterior probabilities (%) and the scale bar the expected substitutions per site. Morphospecies identified by microscopic examination of blood smears are shown.

Four different morphospecies belonging to genus *Plasmodium* and one morphospecies of genus *Haemoproteus* were detected in the blood of examined house sparrows. Morphospecies of parasites which were identified by microscopic examination of blood films are given in Figure 2.

**Figure 3:**
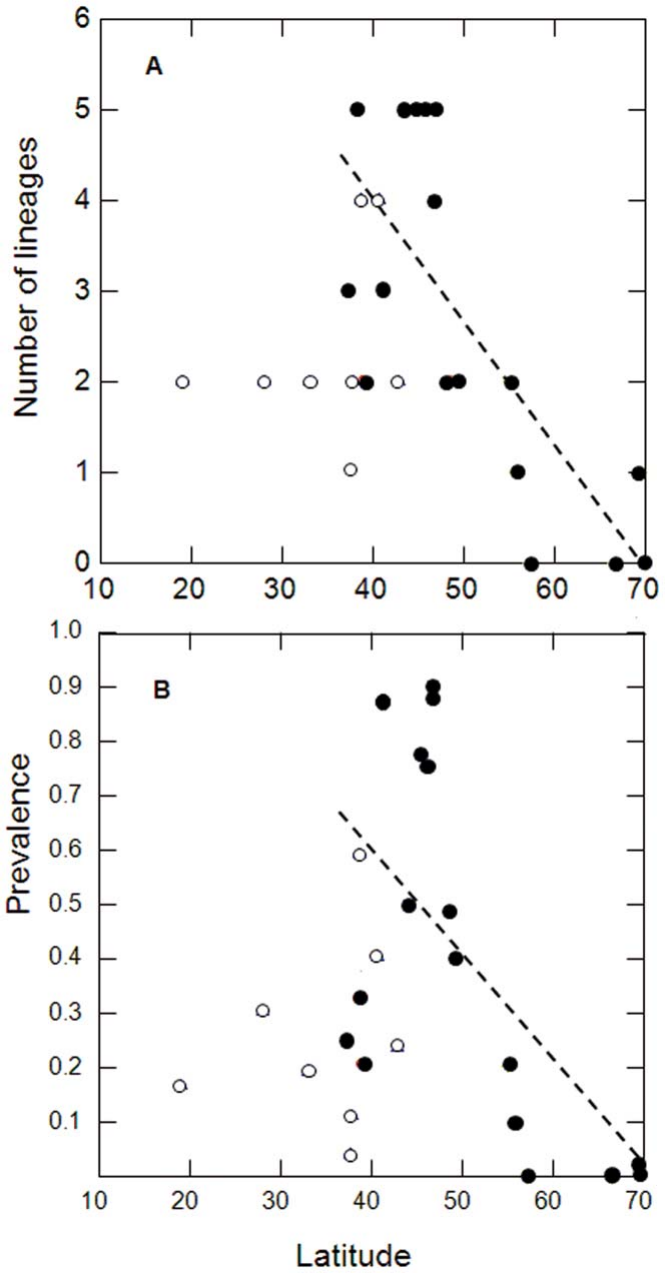
The number lineages (A) and overall prevalence (B) of *Plasmodium* spp. in European (filled circles) and North American (open circles) house sparrows in relation to latitude. The analyses include data from sites where 15 or more sparrows have been sampled. The stippled lines show the significant regression lines for the European sites.

Of the 30 lineages, 13 were recorded in single individuals (Supplementary Table S2). Our statistical analyses of haemosporidian parasites' diversity and distribution focused on the 17 lineages found in two or more individual hosts. *Haemoproteus* parasites (including four lineages of *Haemoproteus passeris*) occurred only at sites around the Mediterranean Sea and in Central Europe (Fig. 2), coinciding with regions having the highest diversity of *Plasmodium* lineages. Within Europe, both prevalence and the diversity of lineages declined significantly towards the north (Fig. 3) and towards the east (Table 1). The latitudinal pattern is consistent both with the spread of house sparrows from south to north accompanied by a loss of parasites [14], [25], and with a reduced diversity of vectors [13] combined with a shorter season for transmission [12]. We did not find any relationship with latitude or longitude within either North America or South America (Table 1).

**Table 1:** Effects of latitude and longitude (multiple regression) on *Plasmodium* spp. prevalence and diversity within Europe, North America and South America.

	Prevalence			Number of Lineages		
	Slope	*t*	*P*	Slope	*t*	*P*
*Europe* *17 sites*						
Latitude	−0.030	4.74	<0.001	−0.17	5.40	<0.001
Longitude	0.016	3.02	0.009	0.68	2.50	0.026
Total model	F_2,14_ = 11.2		<0.001	F_2,14_ = 14.7		0.001
*North America* *8 sites*						
Latitude	0.005	0.55	0.61	0.032	0.48	0.65
Longitude	0.004	0.86	0.43	0.033	0.92	0.40
Total model	F_2,5_ = 0.5		0.62	F_2,5_ = 0.5		0.61
*South America* *9 sites*						
Latitude	−0.001	0.30	0.77	−0.020	0.66	0.53
Longitude	−0.006	1.07	0.32	−0.037	1.02	0.34
Total model	F_2,6_ = 0.8		0.49	F_2,6_ = 1.1		0.50

Only sites with more than 15 screened sparrows were included.

Overall, the mean number of *Plasmodium* lineages differed significantly between American and European regions (F3,31 = 5.85, P = 0.003). Four lineages of *Plasmodium* were recovered from each of North and South America, with fewer lineages (±SE) per site in North America (2.0±0.47) and South America (1.2±0.18) than in SW Europe (3.2±0.60) and SE Europe (3.4±0.56). Moreover, the six dominant American parasite lineages did not occur among house sparrows anywhere in Europe (Fig. 2). The house sparrows in the Americas most likely originated from Europe, as inferred from recorded releases of imported sparrows [16], [18] and high genetic similarity of house sparrows in Europe and North America [33]. Hence, in colonizing the Americas, house sparrows lost the parasites from their native European range and assimilated a new suite of parasites. Therefore, we can reject the Novel Weapons Hypothesis (NWH) to the extent that malaria parasites and related haemosporidians might explain the success of the house sparrow in the Americas.

Elsewhere in the colonized range of house sparrows the pattern is less clear, partly because our data are less complete. The presence of the *P. relictum* lineage SGS1 in New Zealand could indicate that introduced house sparrows did not leave this natural enemy behind and thus its establishment might have been facilitated by a novel weapon. However, at present, it is firmly established whether SGS1 is an native parasite in New Zealand or introduced by house sparrows or any other non-native bird species.

The lineage GRW4 has been found infecting house sparrows at many sites within its introduced range, but it is practically absent from the original range (Supplementary Table S2) except India [22]. It is possible that introduced house sparrows picked up GRW4 during the early phase of colonizing new territories, and that GRW4 functioned as a novel weapon at later stages in the colonization process, for example when colonizing Bermuda, Hawaii, and other islands in the Pacific. Alternatively, it is also possible that other introduced species carried GRW4 with them and the presence of appropriate vectors helped in the establishment of this lineage. The presence of this lineage in parts of its original range may show that not all the enemies were left behind by house sparrows when other parts of the world were colonized. Similar results were found by Ishtiaq et al. [28] in the common myna, where not all comparisons between introduced and native populations were consistent with expectations of the ERH. Although our study covered most of the house sparrow's global distribution, the number of samples from eastern Asia was limited; therefore, parasite lineages infecting house sparrows could have been missed in this area.

**Figure 4:**
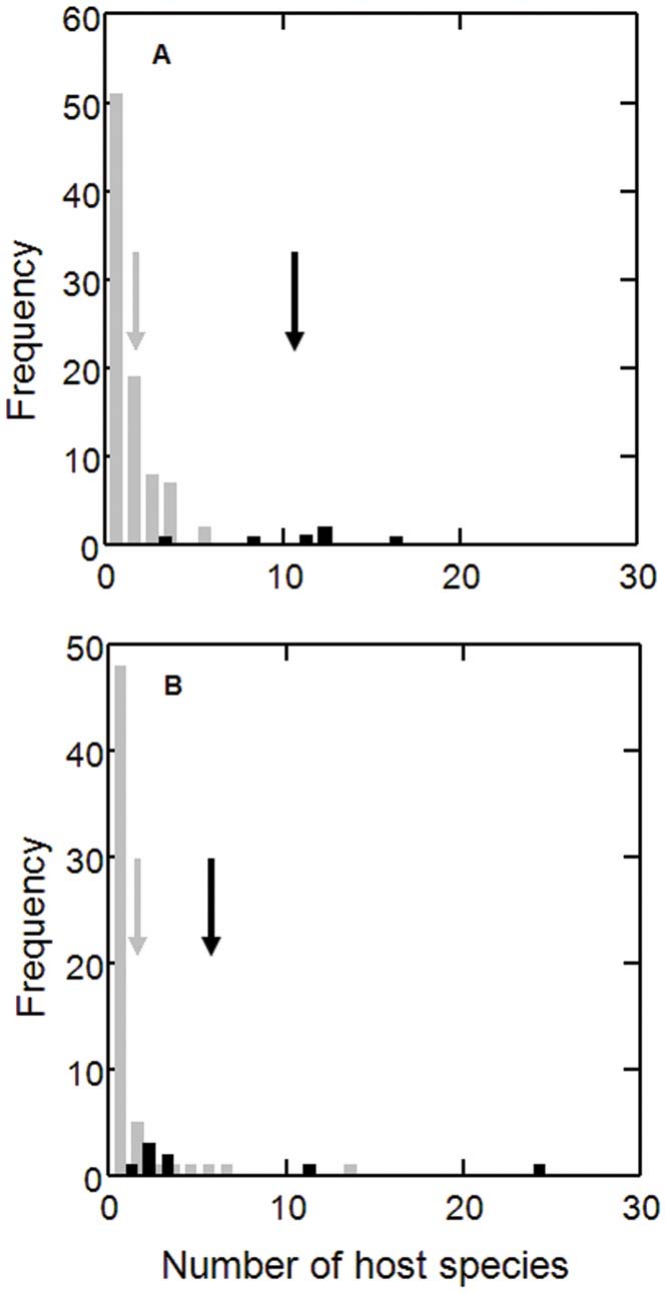
Number of host species in America (A) and Europe (B) for *Plasmodium* spp. lineages infecting (filled bars) and not infecting house sparrows (grey bars). Arrows indicate mean values. The data were extracted from the MalAvi database [15]. Logistic regression (estimate ± SE, P) on the likelihood of observing lineages in house sparrows; number of hosts (0.61±0.19, P = 0.001), America or Europe (−2.17±1.00, P = 0.030) and their interaction term (0.39±0.19, P = 0.043).

All six lineages of malaria parasite found in American house sparrows have previously been identified in other American bird species (MalAvi database [15]). Logistic regression showed that these had significantly (P<0.001) wider host distributions than the 87 lineages recorded in other North American birds but absent from house sparrows (Fig. 4a). Therefore, house sparrows in America have assimilated the generalist parasites of the new continent. The most common of these, *Plasmodium cathemerium* (lineage SEIAUR01) has been recovered from 16 American host species from 9 families [15]. The *Plasmodium* parasites of European house sparrows also had broader host distributions (P = 0.009) than those parasitizing other birds in Europe (Fig. 4b), but the effect was significantly weaker than in the Americas (P = 0.043) as European house sparrows are also infected by parasites with narrow host ranges. It is worth mentioning that four lineages of *Haemoproteus passeris* (Fig. 2) have been reported exclusively in the native range of house sparrows. Although we cannot fully reject the NWH as a factor in the success of the house sparrow outside its native range, the observation that house sparrows do not retain their native parasite fauna in newly colonized regions is consistent with the Enemy Release Hypothesis (ERH).

In further support of the ERH, the average prevalence of *Plasmodium* spp. per site was higher in SW Europe (52.9%±13.9, 5 sites), SE Europe (61.6%±11.1, 6 sites) and the SE Mediterranean area (42.0%±17.0, 4 sites) compared to North America (25.6%±6.2, 8 sites) and South America (13.7%±3.0, 9 sites). We recovered no parasites from populations in the Azores Islands colonized in 1957 [34] (n = 75), Faroe Islands colonized in 1930's [35] (n = 54), and one site in Panama, Central America colonized in 1976 [36] (n = 33). Several less intensively sampled regions of non-native house sparrow populations had lower malaria parasite prevalence than in southern Europe (Kenya, 6.8%; New Zealand, 21.2%). In contrast, the prevalence of *Plasmodium* was exceptionally high (81.4%) in Bermuda (24 cases of GRW4 and 1 of PADOM01). Clearly, the composition of parasite communities differed drastically between the non-native house sparrow populations on continents and islands (which often are free from predators), possibly resulting from different trade-offs between immune function against parasites and predator defence [26]. Alternatively, these differences may show variation in exposure among localities caused by differences in the distribution and/or abundance of different dipteran vector species [37] and variable vector competence [38], [39]. In addition, the presence of strong adaptive immune defences to control novel avian malaria infection in successful colonizing sparrows [40], along with mortality caused by haemosporidians [30], [41] reducing the number of infected birds in our samples could also explain differences in the prevalence of different parasite lineages among localities. Further field studies, ideally combined with experimental research are needed to answer these questions.

The success of the house sparrow in colonizing novel environments is unquestionably linked to the widespread favourable agricultural and urban habitats resulting from human activities. We show that house sparrows in non-native regions posses lower diversity and prevalence of haemosporidian infections than in their native range, consistent with the Enemy Release Hypothesis. That is particularly evident in case of different lineages of *H. passeris*, which are absent from the introduced range (Fig. 2). A striking pattern is that the parasite community of house sparrows in the Americas consist mainly of host generalists. Whether generalist parasites have different impacts than specialist parasites on the survival and reproductive success of hosts is debated [42], [43], but recent studies suggest major differences in transmission success [44] and how they affect host community structure [45]. Detailed comparative studies of house sparrows, their malaria parasites and co-existing host species, in native and non-native ranges, will shed light on these important questions for understanding the impact of invasive species on the biodiversity of the invaded communities.

## Materials and Methods

### Ethical Statement

This study was carried out in strict accordance with current laws of all the countries where the study was performed and following the recommendations of the Guidelines to the Use of Wild Birds in Research (Fair, J., E. Paul, and J. Jones, Eds. 2010. Washington, D.C.: Ornithological Council). All procedures were approved by the University of South Florida IACUC (protocol #W3202), the University of Missouri-St. Louis IACUC (protocols S99-3, 02-18, and 06-02-01) and Swedish Ethical Committee on Animal Experiment (reference M64-05). All efforts were made to minimize handling time and potential suffering of animals. None of the sparrows suffered apparent injury during blood extraction sampling.

### Field work and sampling sites

A total of 1,820 adult or independent juvenile house sparrow blood samples from 58 sites were collected (Fig. 1; Supplementary Table S1) during the breeding seasons 2004–2007. The sparrows were captured when the prevalence of hematozoa among birds is typically highest [12]; in the North hemisphere from February to July and in the South hemisphere: from July to April. Data from India were obtained from Ishtiaq et al. [46]. Blood samples were stored in 500 µl 96% ethanol at room temperature or SET buffer (0.15 M NaCl, 0.05 M Tris, 0.001 M EDTA, pH 8.0) until DNA extraction. Blood films were fixed with methanol in the field and then stained with Giemsa in the laboratory.

### Genetic and microscopic analyses

DNA from the avian blood samples was extracted using a standard chloroform/isoamylalcohol method [47]. Diluted genomic DNA (25 ng/µl) was used as a template in a polymerase chain reaction (PCR) assay for detection of the parasites using a nested-PCR protocol [48]. This methodology is among the most widely used PCR-based protocols for avian haemosporidians. Moreover, it accurately estimates the molecular diversity of the full cytochrome *b* gene and shows consistent results between morphological and molecular analyses [49]. Parasites detected by a positive amplification were sequenced using standard procedures [50]. Amplified fragments were sequenced from 5′end with HaemF. The obtained sequences of 478 bp of the cyt b were edited, aligned and compared in a sequence identity matrix using the program BioEdit [51]. New lineages (sequences not previously published in GenBank) were also sequenced from the reverse end using the primer HaemR2. Sequences differing by at least 1 bp were considered unique lineages. All new DNA sequences have been deposited in GenBank (see Supplementary Table S2).

To identify parasite morphospecies and to link new lineages to them, blood films from the PCR-positive samples were examined microscopically. Blood samples with single infection of corresponding haemosporidian parasite species, as determined both by PCR-based diagnostic and microscopy, were used for this purpose. The recorded parasites (see Fig. 2) were identified to morphospecies according to Valkiūnas [12]. Identifications were done only in sampleswith necessary parasite blood stages for species identification.

### Statistical analyses

Seventy-five samples from 20 sampling localities were randomly chosen to be reanalyzed in order to estimate repeatability. The agreement between these repeated PCR tests of malaria infection was 0.973. The overall reliability as calculated by the kappa statistic [52] was 0.945.

A phylogeny containing the recorded *Plasmodium* and *Haemoproteus* lineages was constructed using Bayesian inference as implemented in MrBayes version 3.1.1. [53]. The phylogeny was constructed with a model of molecular evolution allowing for six substitution rates, a proportion of invariable sites, rate among-site variation in rates of nucleotide substitution drawn from a gamma distribution (GTR+I+G), as selected by AIC criteria in MrModeltest 2.2 [54]. Two simultaneous runs were conducted with a sample frequency of every 100th three over 3 million generations per run. Before constructing a majority consensus tree 25% of the first sampled trees in each run were discarded as burn-in period. Three lineages of *Leucocytozoon* were used as the outgroup (ACCBRE02, DQ177235; GRUS1, DQ847257; SYAT20, DQ847235). The phylogeny was visualized using MEGA 4.0. [55].

Multiple and logistic regression analyses and ANOVAs were carried out in the program SYSTAT 10.0 [56]. Information of host distribution of lineages was extracted from the MalAvi database (http://dx.doi.org/10.1111/j.1755-0998.2009.02692.x) [15].

## Supporting Information

Table S1 Sampling sites of house sparrows, geographic grouping, sample size, number of positives, overall prevalence of infection and number of mixed infections of haemosporidian lineages.
(RTF)

Table S2 Lineage names, parasite genus (H = *Haemoproteus*, P = *Plasmodium*), GenBank accession numbers and number of infections per country; Italy (It), France (Fr), Spain (Sp), Bulgaria (Bu), Czech Republic (Cz), Romania (Ro), Russia (Ru), Lithuania (Lit), Norway (No), Sweden (Sw), Egypt (Eg), Israel (Is), Turkey (Tk), Mexico (Mx), USA (USA), Argentina (Ar), Brazil (Br), Bermuda (Ber), Kenya (Ken), India (Ind) and New Zealand (NZ). Excluded from the Table are samples from four sites without haemosporidian infections (Denmark, n = 17; Faroe Islands, n = 54; Azores, n = 75; Panama, n = 33).
(RTF)
