# Supplementary material for: Diversity, Loss, and Gain of Malaria Parasites in a Globally Invasive Bird
Source: PLoS One. 2011 Jul 11;6(7):e21905. doi: 10.1371/journal.pone.0021905 (PMC3136938; doi:10.1371/journal.pone.0021905)
Supplement: Table S1 — Sampling sites of house sparrows, geographic grouping, sample size, number of positives, overall prevalence of infection and number of mixed infections of haemosporidian lineages. (RTF) [file pone.0021905.s001.rtf]

Supporting Information

Supplementary Table S1.
Sampling site	
Latitude / Longitude	Region	N	
No. infected	
Prevalence (%)	No. mixed infections	
ITALY, Caserta	41 / 14.2	SW EUROPE 	48	40	83.3	2	
FRANCE, Chizé	46.1 / - 0.2	SW EUROPE 	166	109	65.7	16	
FRANCE, Paris	48.5 / 2.2	SW EUROPE 	66	37	56.1	0	
SPAIN, Badajoz	38.9 / - 6.9	SW EUROPE 	126	93	73.8	11	
SPAIN, Ciudad Real	39.0 / -3.9	SW EUROPE 	29	7	24.1	0	
SPAIN, Granada	37.2 / - 3.6	SW EUROPE 	36	8	22.2	1	
BULGARIA, Kalimok	44.0 / 26.4	SE EUROPE 	36	19	52.8	1	
CZECH REP., Veseli nad Luznici	49.2 / 14.7	SE EUROPE 	50	20	40.0	0	
ROMANIA, Cojocna	46.5 / 23.5	SE EUROPE 	33	23	69.7	7	
ROMANIA, Cluj Napoca	46.5 / 23.3	SE EUROPE 	31	25	80.6	3	
ROMANIA, Vãcãreni	45.2 / 28.1	SE EUROPE 	40	28	70.0	3	
RUSSIA, Diakovka	50.7 / 46.8	SE EUROPE 	7	7	100	2	
RUSSIA, Krasn, Kut	50.9 / 47.0	SE EUROPE 	3	2	66.7	0	
DENMARK, Kraghede	57.2 / 10.0	N EUROPE 	17	0	0	0	
LITHUANIA, Curonian Spit	55.1 / 20.7	N EUROPE 	49	10	20.4	0	
NORWAY, Alta	69.9 / 29.3	N EUROPE 	35	0	0	0	
NORWAY, Hestmannøy	66.5 / 12.8	N EUROPE 	42	0	0	0	
NORWAY, Pasvik	69.4 / 30.0	N EUROPE 	46	1	2.2	0	
SWEDEN, Skåne	55.7 / 13.3	N EUROPE 	20	2	10.0	0	
EGYPT, Luxor	25.7 / 32.6	SE MEDITERR.	34	29	85.3	1	
ISRAEL, Sede Boger Campus	30.8 / 34.8	SE MEDITERR.	42	5	11.9	0	
TURKEY, Antalya	37.0 / 30.5	SE MEDITERR.	34	16	47.1	3	
TURKEY, Yatagan	37.3 / 28.1	SE MEDITERR.	33	16	48.5	1	
AZORES, Sao Miguel	37.7 / - 25.5	N ATLANTIC 	75	0	0	0	
BERMUDA, Bermuda	32.3 / - 64.7	N ATLANTIC 	27	21	77.8	1	
FAEROE ISLANDS, Torshavn	62.0 / - 6.8	N ATLANTIC 	54	0	0	0	
MEXICO, Mexico City	19.0 / - 99.0	N AMERICA 	18	2	11.1	1	
USA, Arizona	33.0 / -112.0	N AMERICA 	26	5	19.2	0	
USA, Colorado	40.6 / - 104.8	N AMERICA 	42	17	40.5	0	
USA, Connecticut	41.1 / -73.3	N AMERICA 	1	0	0	0	
USA, Kentucky	37.8 / -84.2	N AMERICA 	36	4	11.1	0	
USA, Michigan	42.8 / - 84.6	N AMERICA 	21	5	23.8	0	
USA, Sausalito, California	37.6 / -122.3	N AMERICA 	25	2	8.0	0	
USA, St. Louis, Missouri	38.6 / - 90.2	N AMERICA 	71	39	54.9	3	
USA, Tampa, Florida	28.0 / - 82.0	N AMERICA 	33	10	30.3	0	
PANAMA, Colón	9.0 / - 79.0	C AMERICA	33	0	0	0	
ARGENTINA, Gualeguaychú	- 33.0 / - 58.5	S AMERICA 	9	6	77.8	0	
BRAZIL, Belém	- 1.4 / - 48.5	S AMERICA 	24	0	0	0	
BRAZIL, Cáceres	- 16.1 / - 57.7	S AMERICA 	28	9	32.1	0	
BRAZIL, Campo Grande	- 20.5 / - 54.6	S AMERICA 	22	2	9.1	0	
BRAZIL, Canela	- 29.4 / - 50.8	S AMERICA 	19	3	15.8	0	
BRAZIL, Canoas	- 29.9 / - 51.2	S AMERICA 	5	2	40.0	0	
BRAZIL, Uberlândia	- 18.9 / - 48.3	S AMERICA 	23	2	8.7	0	
BRAZIL, Gurupi	- 11.7 / - 49.1	S AMERICA 	10	1	10.0	0	
BRAZIL, Niteroí	- 22.9 / - 43.1	S AMERICA 	24	3	12.5	0	
BRAZIL, Palmas	- 10.2 / - 48.4	S AMERICA 	15	4	26.7	0	
BRAZIL, Recife	- 8.0 / - 34.9	S AMERICA 	26	2	7.7	0	
BRAZIL, Salvador	- 13.0 / - 38.5	S AMERICA 	18	2	11.1	0	
KENYA, Nairobi	- 1.0 / 37.0	AFRICA 	44	3	6.8	0	
INDIA, Assam	26.3 / 95.9	S ASIA 	6	1	16.7	0	
INDIA, Meerut	28.9 / 77.7	S ASIA 	11	3	27.3	0	
INDIA, Ponjore	29.1 / 76.1	S ASIA 	4	1	25.0	0	
NEW ZEALAND, Benneydale	- 38.5 / 175.3	NEW ZEALAND 	4	0	0	0	
NEW ZEALAND, Drury	- 37.2 / 174.9	NEW ZEALAND 	7	2	28.6	0	
NEW ZEALAND, Havelock North	- 39.7 / 176.9	NEW ZEALAND 	6	0	0	0	
NEW ZEALAND, Little Barrier Is.	- 36.2 / 175.1	NEW ZEALAND 	21	4	19.0	0	
NEW ZEALAND, Palmerston North	- 40.4 / 175.6	NEW ZEALAND 	4	1	25.0	0	
NEW ZEALAND, Tiritiri Matangi Is.	- 36.6 / 174.9	NEW ZEALAND 	5	3	60.0	0	
