# Supplementary material for: Diversity, Loss, and Gain of Malaria Parasites in a Globally Invasive Bird
Source: PLoS One. 2011 Jul 11;6(7):e21905. doi: 10.1371/journal.pone.0021905 (PMC3136938; doi:10.1371/journal.pone.0021905)
Supplement: Table S2 — Lineage names, parasite genus (H = Haemoproteus, P = Plasmodium), GenBank accession numbers and number of infections per country; Italy (It), France (Fr), Spain (Sp), Bulgaria (Bu), Czech Republic (Cz), Romania (Ro), Russia (Ru), Lithuania (Lit), Norway (No), Sweden (Sw), Egypt (Eg), Israel (Is), Turkey (Tk), Mexico (Mx), USA (USA), Argentina (Ar), Brazil (Br), Bermuda (Ber), Kenya (Ken), India (Ind) and New Zealand (NZ). Excluded from the Table are samples from four sites without haemosporidian infections (Denmark, n = 17; Faroe Islands, n = 54; Azores, n = 75; Panama, n = 33). (RTF) [file pone.0021905.s002.rtf]

Supporting Information

Supplementary Table S2.

					Country site	
Lineage	Genus	GenBank#	Total		It	Fr	Sp	Bu	Cz	Ro	Ru	Lit	No	Sw	Eg	Is	Tk	Mx	USA	Ar	Br	Ber	K	In	NZ	
ALBRE01	H	AF465571	1		-	-	-	-	-	-	-	-	-	-	-	-	-	-	-	-	-	-	-	1	-	
COLL1	P	AY831747	20		-	10	3	-	-	7	-	-	-	-	-	-	-	-	-	-	-	-	-	-	-	
DENPET3	P	AY640137	4		-	-	-	-	-	-	-	-	-	-	-	-	-	-	-	-	4	-	-	-	-	
GASAN01	P	AY172847	1		-	-	-	-	-	-	-	-	-	-	-	-	-	-	1	-	-	-	-	-	-	
GRW1	H	AF254964	1		-	1	-	-	-	-	-	-	-	-	-	-	-	-	-	-	-	-	-	-	-	
GRW4	P	AF254975	69		-	-	-	-	-	-	-	-	-	-	-	-	-	1	28	6	2	21	2	4	5	
GRW6	P	DQ368381	4		-	-	-	3	-	-	-	-	-	-	-	-	-	-	-	-	-	-	-	-	1	
GRW11	P	AY831748	42		12	20	9	1	1	21	3	4	-	-	-	-	6	-	-	-	-	-	-	-	-	
PADOM01	P	DQ058611	18		2	4	8	1	-	2	-	-	-	-	-	-	-	-	-	-	-	1	-	-	-	
PADOM02	P	DQ058612	10		-	1	2	-	-	5	-	-	-	-	1	-	1	-	-	-	-	-	-	-	-	
PADOM03	H	DQ058614	4		-	4	-	-	-	-	-	-	-	-	-	-	-	-	-	-	-	-	-	-	-	
PADOM05	H	HM146898	76		-	-	58	-	-	-	7	-	-	-	-	-	11	-	-	-	-	-	-	-	-	
PADOM06	P	GU065647	1		-	-	-	1	-	-	-	-	-	-	-	-	-	-	-	-	-	-	-	-	-	
PADOM08	P	GU065648	1		-	-	1	-	-	-	-	-	-	-	-	-	-	-	-	-	-	-	-	-	-	
PADOM09	P	AF069611	20		-	-	-	-	-	-	-	-	-	-	-	-	-	-	-	-	20	-	-	-	-	
PADOM11	P	HM146899	8		-	-	-	-	-	-	-	-	-	-	-	-	-	-	6	-	2	-	-	-	-	
PADOM15	H	HM146900	1		-	-	1	-	-	-	-	-	-	-	-	-	-	-	-	-	-	-	-	-	-	
PADOM16	P	HM146901	18		-	-	-	-	-	-	-	-	-	-	18	-	-	-	-	-	-	-	-	-	-	
PADOM17	P	HM146902	1		-	-	-	-	-	-	-	-	-	-	-	-	-	-	-	-	1	-	-	-	-	
PADOM18	P	GU065649	1		-	-	-	-	-	1	-	-	-	-	-	-	-	-	-	-	-	-	-	-	-	
PADOM19	P	HM146903	1		-	-	-	-	-	-	-	-	-	-	-	-	-	-	-	-	1	-	-	-	-	
PADOM20	P	HM146904	1		-	-	1	-	-	-	-	-	-	-	-	-	-	-	-	-	-	-	-	-	-	
PADOM21	P	HM146905	1		-	-	-	-	-	1	-	-	-	-	-	-	-	-	-	-	-	-	-	-	-	
PADOM22	H	GU065650	2		-	-	2	-	-	-	-	-	-	-	-	-	-	-	-	-	-	-	-	-	-	
PAHIS01	H	GU065651	7		-	-	2	2	-	1	-	-	-	-	-	-	2	-	-	-	-	-	-	-	-	
SEIAUR01	P	DQ838988	47		-	-	-	-	-	-	-	-	-	-	-	-	-	2	45	-	-	-	-	-	-	
SGS1	P	AF495571	312		28	122	33	12	19	51	1	6	1	2	11	5	16	-	-	-	-	-	1	-	4	
SISKIN1	H	AY393806	1		-	-	-	-	-	-	-	-	-	-	-	-	-	-	1	-	-	-	-	-	-	
WW3	P	AF495577	3		-	-	-	-	-	-	-	-	-	-	-	-	-	-	3	-	-	-	-	-	-	
ZEMAC1	P	AY099032	1		-	-	-	-	-	-	-	-	-	-	-	-	-	-	1	-	-	-	-	-	-	
																										
Total infected					42	162	120	20	20	89	11	10	1	2	30	5	36	3	85	6	30	22	3	5	10	
tested					48	232	191	36	50	104	10	49	123	20	34	42	67	18	255	9	214	27	44	21	47	
															
															
